# Granular Nanofiber‐Hydrogel Composite‐Programmed Regenerative Inflammation and Adipose Tissue Formation

**DOI:** 10.1002/adhm.202403094

**Published:** 2024-11-24

**Authors:** Jiayuan Kong, Zhi‐Cheng Yao, Jessica L. Stelzel, Yueh‐Hsun Yang, Jeffrey Chen, Hexiang Feng, Collin Schmidt, Chi Zhang, Kedar Krishnan, Long Chen, Jingwen Pan, Kailei Ding, Yining Zhu, Xiaowei Li, Joshua C. Doloff, Hai‐Quan Mao, Sashank K. Reddy

**Affiliations:** ^1^ Department of Materials Science and Engineering Johns Hopkins University Baltimore MD 21218 USA; ^2^ Translational Tissue Engineering Centre Johns Hopkins University School of Medicine Baltimore MD 21213 USA; ^3^ Institute for NanoBioTechnology Johns Hopkins University Baltimore MD 21218 USA; ^4^ Department of Biomedical Engineering Johns Hopkins University School of Medicine Baltimore MD 21205 USA; ^5^ Department of Plastic and Reconstructive Surgery Johns Hopkins School of Medicine Baltimore MD 21287 USA; ^6^ Department of Orthopaedics Guizhou Provincial People's Hospital Guiyang Guizhou 550000 P. R. China; ^7^ Department of Surgery Washington University School of Medicine St. Louis MO 63110 USA

**Keywords:** biomaterials, hydrogels, regenerative inflammations, soft tissue restoration

## Abstract

The interplay between biomaterials and host immune responses critically determines outcomes in tissue restoration. Recent studies suggest that physicochemical properties of materials can dictate pro‐regenerative versus pro‐fibrotic responses and have begun to define the key immune cell types and signals governing these divergent effects. This emerging understanding enables the engineering of regenerative biomaterials capable of functional restoration in situ. An injectable nanofiber‐hydrogel composite (NHC) microparticles are designed and constructed from cross–linked electrospun collagen nanofiber fragments surface‐bonded to the hyaluronic acid hydrogel network via covalent conjugation during the cross–linking process. The collagen nanofiber fragments, acting as the structural reinforcement component, increased the overall storage modulus of the NHC to a level comparable to native soft tissues while maintaining a sufficiently high degree of porosity of the hydrogel phase to allow host cell infiltration following subcutaneous injection of the NHC microparticles. More importantly, the NHC promoted macrophage/monocyte infiltration, migration, and spreading, sustained cell recruitment over time, and enhanced the proangiogenic effect and recruitment of PDGFRα^+^ perivascular progenitor cells, leading to extensive adipose tissue remodeling. This study demonstrates the regenerative potential of the injectable NHC microgels as an off‐the‐shelf solution for devastating soft tissue losses.

## Graphical abstract

An injectable collagen nanofiber‐hyaluronic acid hydrogel composite elicits a pro‐regenerative inflammation response characterized by controlled and sustained macrophage recruitment and polarization, leading to angiogenesis and adipose tissue remodeling following subcutaneous injection in rats. This composite allows for better host tissue integration and soft tissue remodeling compared to traditional hydrogels, offering a promising off‐the‐shelf solution for patients suffering from soft tissue defects.

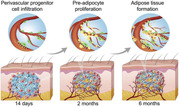

## 1 Introduction

Adipose tissue loss from trauma, oncologic resection, and aging is a source of considerable morbidity.^[^ ^1^ ^]^ Current methods for adipose tissue restoration, such as flaps and lipotransfer, require invasive surgery and are hampered by variable outcomes.^[^ ^2^ ^]^ Prosthetic implants can also replace adipose tissue volume, but these lack native tissue feel and are plagued by poor biocompatibility, leading to fibrosis, infection, and device failure.^[^ ^3^ ^]^ Adipose tissue engineering using biomaterial scaffolds has emerged as an alternative to these autologous and prosthetic approaches. Naturally derived biomaterials, such as collagen and other extracellular matrix (ECM) components, have been utilized as scaffolding materials for adipose tissue reconstruction.^[^ ^4^ ^]^ While these materials demonstrate good biocompatibility, they engender limited adipose tissue remodeling in the absence of additional chemical or biological cues.^[^ ^5^ ^]^

Injectable hydrogels are widely used in the repair and restoration of soft tissues due to their extensive tunability and ability to mimic the microenvironment of native tissue.^[^ ^6^ ^]^ Hydrogels with storage moduli (*G*’) comparable to those of native soft tissue can achieve better volume and shape retention after implantation.^[^ ^7^ ^]^ Among those, hydrogels prepared from hyaluronic acid (HA), a naturally occurring polysaccharide, have been widely used as fillers and foams for soft tissue restoration due to its biocompatibility, biodegradability, and comparable physical properties when cross–linked to those of native soft tissues.^[^ ^8^ ^]^ However, to achieve the necessary mechanical properties for durable soft tissue restoration in vivo, HA and other traditionally synthesized hydrogels must be densely cross–linked, which in turn limits the infiltration of host cells.^[^ ^9^ ^]^ As expected, there has been a minimum degree of neo‐tissue formation in these HA hydrogels. Following hydrogel injection, monocytes and macrophages are among the early responders that emerge as key regulators coordinating host tissue responses. Polarization of these macrophages across a spectrum of phenotypes initiates the programming of pro‐fibrotic or pro‐regenerative responses.^[^ ^10^ ^]^ The properties of hydrogel matrices themselves contribute to macrophage infiltration and programming in acute and chronic inflammation.^[^ ^11^ ^]^ For example, polarization between pro‐fibrotic M1 and pro‐regenerative M2 phenotypes can be modulated by changing the mechanical properties of hydrogels and correlates to the degree of the subsequent reconstructive remodeling.^[^ ^12^ ^]^ While increasing hydrogel pore size and reducing mechanical stiffness can promote host macrophage infiltration and conditioning, the tradeoff is reduced structural integrity with poor tissue restoration in vivo. Further, with hydrogels alone in the absence of exogenous bioactive factors, macrophage activity is not sustained during hydrogel degradation, leading to limited tissue remodeling.^[^ ^13^ ^]^

To improve macrophage recruitment and polarization while retaining structural integrity, we previously developed a chemically defined nanofiber‐hydrogel composite (NHC) by incorporating electrospun poly‐ε‐caprolactone (PCL) nanofiber fragments into HA hydrogel through covalent interfacial bonding.^[^ ^14^ ^]^ This enabled sufficient mechanical strength for in vivo soft tissue volume replacement immediately following injection while maintaining the porosity required for cell infiltration and proangiogenic response. By replacing the PCL nanofibers with an electrospun cross–linked Type I bovine collagen nanofiber fragments distributed throughout a macroporous HA hydrogel using divinyl sulfone (DVS) as a cross–linker,^[^ ^15^ ^]^ here we create a collagen nanofiber‐based NHC using two natural biopolymers, collagen, and HA and examine the biostimulatory potential of the NHC that lead to macrophage recruitment and programming for matrix‐mediated angiogenesis and adipose tissue formation.

## 2 Results

### 2.1 Preparation and Characterization of Collagen Nanofiber‐HA Hydrogel Composite (NHC)

**Figure 1:**
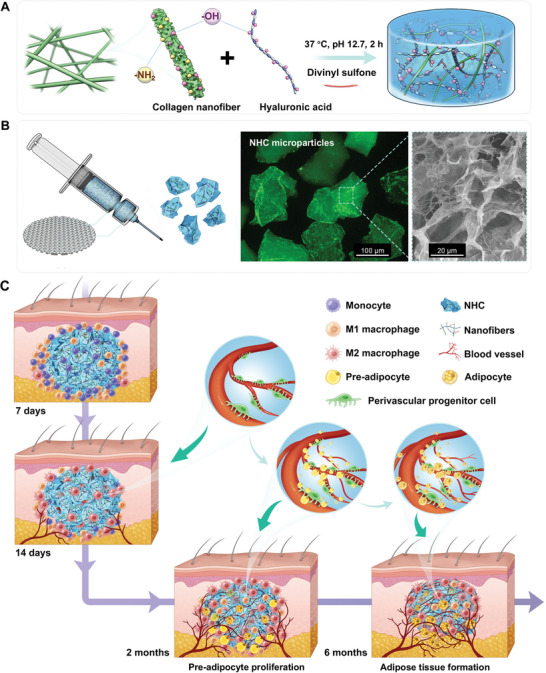
Synthesis of the collagen nanofiber‐based NHC and schematic illustration of the NHC microparticle‐mediated regenerative inflammation, angiogenesis, and adipose tissue remodeling. A) NHC is synthesized by cross–linking collagen fiber fragments with HA by divinyl sulfone (DVS) at 37 °C, pH12.7 for 2 h. B) NHC microparticles are formed by mechanical fragmentation through metal screens with a defined pore size; an immunofluorescence image showing granular NHC with irregular shapes and sizes ranging from 200 to 250 µm capable of passing through a 27‐G needle; a scanning electron microscopy (SEM) image showing nanofibers distributed in the dried hydrogel matrix. C) A schematic showing a series of inflammation and angiogenic responses modulated by the NHC microenvironment toward tissue remodeling following subcutaneous injections.

The new NHC construct consists of three components: an HA network, bovine type I collagen nanofibers, and DVS as a cross–linker (**Figure** **1**A). The collagen nanofibers were prepared by electrospinning an 8 w/v% collagen solution in hexafluoro‐2‐propanol (HFIP) at a voltage of 25 kV and flow rate of 5 mL h^−1^, resulting in a fiber diameter ≈600 nm (Figure S1A, Supporting Information). The cross–linking of HA with DVS was optimized at a lower pH of 12.7 to minimize the degradation of HA chains and collagen fibers during gelation (Figure S2, Supporting Information). The upper bound of storage modulus was achieved with 2 h of cross–linking at 37 °C. (Figure S2, Supporting Information). Collagen nanofibers were then introduced to the cross–linking HA network to form a covalently linked composite with interfacial bonding. This composite was then fragmented through a metal mesh twice to generate irregularly shaped hydrogel microparticles (Figure 1B; Figure S3A, Supporting Information). The optical microscope image and SEM images (Figure S3B, Supporting Information) showed that the nanofiber fragments were well‐distributed and entangled within the HA hydrogel phase. In addition, the SEM images of HA hydrogels revealed the microstructural differences between the NHCs and HAs (Figure S2B, Supporting Information).

The cross–linked HA and NHC were then fragmented into granular particles with amorphous shapes (Figure 1B) that were able to easily pass through a 27‐G needle (Figure S2A, Supporting Information) — a key design requirement for minimally invasive soft tissue replacement. To quantify the reinforcement effect, the storage modulus of the HA hydrogel phase (*G*_0_′), the overall G′ of the NHC, and the *G*′ of a hydrogel‐nanofiber mixture without interfacial bonding were measured. In rheological tests with fiber loading densities of 1 to 3 w/v%, the *G*′ of the composite was 1.5 to 3 times higher than that achieved without interfacial bonding, with the difference increasing as a function of both fiber loading and cross–linker concentration (Figure S2E,F, Supporting Information). The stiffness enhancement due to the addition of fibers was also dependent on the average fiber length and cross–linking density with fibers between 40 and 100 µm yielding the largest stiffness enhancement (Figure S2G, Supporting Information), and the difference in enhancement was inversely related to cross–linker concentration. This suggests that the differences in stiffness enhancement may be due to the different surface areas of the nanofibers at different lengths. Fibers with an average length below 40 µm have a lower average surface area and, thus, did not show significant enhancement in mechanical properties. On the contrary, long fibers (> 100 µm) with a larger surface area may form substantial cross–links among themselves instead of reacting with the HA network, preventing the stiffness enhancement for the NHC matrix. Typically, we used fiber fragments with an average length of 40 to 100 µm for in vivo studies. To measure the lengths of the fragmented fibers, we dispersed them in ethanol and imaged them with an optical microscope under a 40× objective lens (Figure S1B, Supporting Information) and then quantified the lengths using ImageJ (Figure S1C, Supporting Information). The quantitative analysis suggests that the average length of the fiber fragments in the tested NHC was 47.4 ± 2.6 µm (Figure S1D, Supporting Information).

As a result, these tuning steps allowed the generation of NHC with *G*′ in the range of 200 – 1500 Pa, encompassing the stiffnesses of diverse native soft tissues (Figure S2H, Supporting Information). The cross–linked NHCs and HA hydrogels were then dialyzed and swelled against phosphate buffer and then reached a final *G*′ range of 100 – 1000 Pa (Figure S2I, Supporting Information). To mimic the adipose tissue microenvironment, HA controls and composites were fabricated with comparable rheologic parameters (*G*′ = 250 Pa and *G*_0_′ = 100 Pa) (Figure S2I, Supporting Information). The materials were then particulated into granular microgels of ≈150 µm diameter for both HA hydrogels and NHC and sterilized by autoclave, with no loss of storage modulus (Figure S2J,K, Supporting Information).

### 2.2 Analysis of Matrix Biocompatibility and Improvement of Cell Adhesion and Migration

Collagen nanofibers contain ample cell adhesion ligands that support cell adhesion and migration through the HA hydrogel.^[^ ^16^ ^]^ In this study, we seek to investigate how collagen nanofibers would enhance the cell activities in the NHC. To compare the capacity of the NHC microparticles versus the hydrogel controls to support cell attachment and migration, human adipose‐derived stem cells (hADSCs) and human umbilical vein endothelial cells (HUVECs) were cultured in three fragmented microgels: 100‐Pa HA, 250‐Pa HA, and 250‐Pa NHC. The 100‐Pa HA and 250‐Pa NHC have similar cross–linking densities as both were prepared using the same HA concentration and cross–linker‐to‐HA ratio and, therefore, have similar pore sizes based on the SEM imaging analysis (Figures S2B and S3B, Supporting Information). As a result of the nanofiber reinforcement effect, the shear storage modulus (*G*’) of the NHC was increased to 250 Pa. The 250‐Pa HA microgel was included in this study as a stiffness‐matched hydrogel control for the 250‐Pa NHC; it was prepared using a higher cross–linker to HA ratio (Figure S2I, Supporting Information), corresponding to smaller pore sizes (Figure S2B, Supporting Information).

The viability of both hADSCs and HUVECs was ≈90% for all three materials and did not significantly decrease on Days 4 and 7 compared to Day 1 (ns, *p* > 0.05; Figure S4A,B, Supporting Information). The attachment of hADSCs and HUVECs in a 2D culture system was improved in 250‐Pa NHC at 4 and 24 h after cell seeding, with the difference minimized after 48 h (Figure S4C,D, Supporting Information). To evaluate any enhancement of cell migration conferred by the nanofiber component, hADSCs were cultured on the 100‐Pa HA hydrogel or a collagen fiber mat placed at the bottom of a 96‐well plate. Substantial cell sprouting and spreading/migration were observed when hADSCs were cultured on a collagen nanofiber mat. In contrast, hADSCs cultured on a 100‐Pa HA hydrogel with the same pore size as the 250‐Pa NHC formed clusters and failed to migrate through the pores of the hydrogel (Figure S4E, Supporting Information). These results indicate that the collagen nanofibers are capable of supporting cell adhesion, migration, and spreading, whereas the 100‐Pa and 250‐Pa HA hydrogels do not provide sufficient adhesion cues to permit migration.

**Figure 2:**
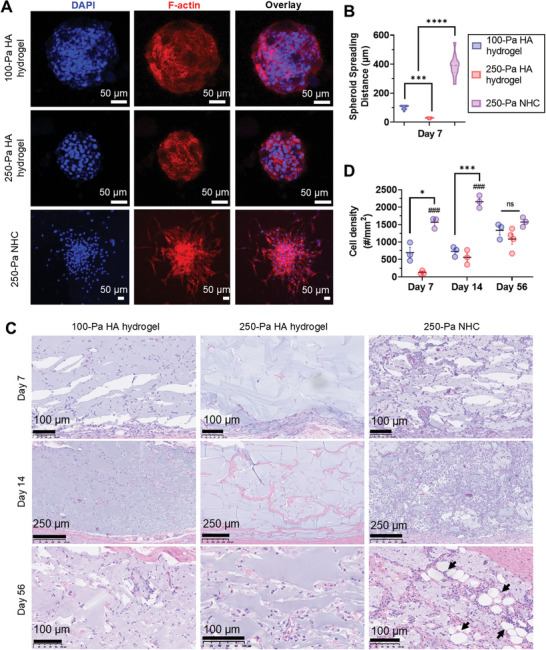
Enhanced cell migration and infiltration mediated by NHC in vitro and in vivo. A) Cell migration and spreading inside the 250‐Pa NHC compared with the 100‐ and 250‐Pa HA visualized by cell spreading from the hADSC spheroids seeded inside the NHC for 7 days (*n =* 9). Scale bars = 50 µm. Cell morphology was visualized by staining for actin with Alexa Fluor 568 Phalloidin shown in red. Cell nuclei were stained with DAPI shown in blue. B) Comparative analysis of hADSC spreading distance from the cell spheroids seeded in the NHC or HA hydrogels with matching stiffness (*G*’ = 250 Pa) or pore size (*G*’ = 100 Pa). C) H&E staining images of the 100‐ and 250‐Pa HA hydrogels and 250‐Pa NHC samples harvested on Days 7, 14, and 56 after *s.c*. injection (*n =* 3). Scale bar = 100 µm. Black arrows indicate potential adipocyte formation. D) Quantitative measurements of cellular infiltration of the 100‐ and 250‐Pa HA hydrogels and 250‐Pa NHC on Days 7, 14, and 56. Statistical significance was calculated by one‐way ANOVA with Dunnett's post hoc test. The asterisk indicates the comparison between each material group. ns*, p* > 0.05, *p < 0.05, **p < 0.01, ***p < 0.001, ****p < 0.0001. Data are presented as means ± SEM.

We next examined cell migration behavior in a 3D culture system where hADSC spheroids were encapsulated in 100‐Pa HA control and 250‐Pa HA (*G*_0_' = 100 Pa) for 7 days.^[^ ^17^ ^]^ No significant cell migration was observed in the 100‐Pa HA control (**Figure** **2**A,B), consistent with previous studies showing limited cell migration in HA hydrogels.^[^ ^14b^ ^]^ In contrast, cell spheroids in 250‐Pa NHC showed extensive sprouting behavior (Figure 2A). Taken together, these studies suggest that collagen nanofibers encourage cell migration through the porous composite structure.

### 2.3 Prolonged Bio‐Stimulatory Effect and Shape Retention of the NHC

To investigate whether the NHC could promote soft tissue volume restoration and regenerative inflammation in vivo, NHC was compared to HA controls in a rodent subcutaneous injection model, with 200 µL of NHC (*G*' = 250 Pa, *G*_0_' = 100 Pa) and 200 µL of HA hydrogels (*G*’ = 100 Pa and 250 Pa) injected in the Sprague‐Dawley (SD) rats. The volume retention of the materials was measured by caliper on Days 7, 14, 56, and 180. After initial swelling, all three groups maintained a volume of ≈300–400 µL until Day 56 (Figure S5A,B, Supporting Information). On Day 180, the volume of the NHC was comparable to the 250‐Pa HA hydrogel control and superior to the 100 Pa HA hydrogel (*p* > 0.05; Figure S5A). Histological analysis demonstrated increased cell infiltration in the NHC on Day 7 compared to both hydrogel controls (*p* < 0.05 compared to 100‐Pa HA hydrogel, and *p* < 0.01 compared to 250‐Pa HA hydrogel), and this infiltration continued to increase on Day 14; Figure 2C,D). By contrast, host cells remained at the periphery of HA hydrogel controls with minimal migration into the injected tissue (Figure 2C). Based on the morphology of the infiltrated cells in the histological images and the nature of foreign body response, the recruited cells are primarily monocytes and macrophages, as subsequently confirmed with immunostaining.^[^ ^18^ ^]^ The faster cell migration into the NHC matrix and the 100‐Pa HA microgel is likely due to the larger pore sizes. The higher number of infiltrated cells observed in the 250‐Pa NHC was largely due to the better cell adhesion property of the collagen nanofibers, facilitating cell migration and spreading.

Strikingly, a significant presence of vasculature and immature adipocytes was noted in the NHC on Day 56, with a paucity of vascularized adipose tissue in HA controls (Figure 2C). Cell density analysis on Day 56 revealed that the cell numbers within the 250‐Pa NHC injection dropped to ≈1500 cells mm^−2^ and did not differ significantly from the 100‐Pa HA control (ns, *p* > 0.05; Figure 2D). While the number of cells on Day 56 was not significantly different, the earlier infiltration into NHC and evident adipose tissue remodeling suggest that NHC promotes a distinct, pro‐regenerative remodeling response.

### 2.4 Macrophage Recruitment and Programming

**Figure 3:**
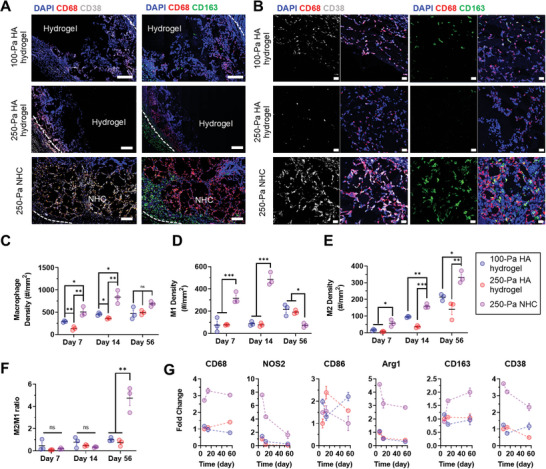
Enhanced infiltration of host macrophages into NHC and effect of NHC on conditioning of macrophage phenotypes. (A, B) Tile‐scans A) and zoomed‐in Z‐stack B) immunofluorescence images to show enhanced CD68^+^ pan‐macrophage (red), CD38^+^ M1 macrophage (gray), and CD163^+^ M2 macrophage (green) infiltration and expression on Day 7. Scale bars, tile‐scans = 200 µm, Z‐stack images = 50 µm. (C–F) Quantitative of the density of infiltrated C) CD68^+^ pan‐macrophages, D) CD38^+^ M1 macrophage, E) CD163^+^ M2 macrophage, and F) M2/M1 ratio on Days 7, 14, and 56 by analyzing the tile‐scan images (*n =* 3). G) Gene expression level of macrophage‐related genes (CD68, Nos2, CD86, Arg1, CD163, and CD38). Expression is normalized to Day 7 250‐Pa HA. Samples were pooled together and then tested in triplicate (*n =* 3). Statistical significance was calculated by two‐way ANOVA with Dunnett's post hoc test. The asterisk indicates the comparison between selected groups. ns, *p* > 0.05, *p < 0.05, **p < 0.01, ***p < 0.001, ****p < 0.0001. Data are presented as means ± SEM.

We next examined the mechanisms behind early tissue remodeling and soft tissue formation in NHC. Given the previously defined roles of macrophages in orchestrating the tissue responses to hydrogels and composites,^[^ ^10a,c^ ^]^ we first turned our attention to macrophage infiltration and polarization. Using markers CD68, CD38, and CD163, we identified pan‐macrophages, M1‐like macrophages, and M2‐like macrophages, respectively, and used tile scan imaging to quantitatively analyze the densities of different subtypes of macrophages.^[^ ^19^ ^]^ By Day 7, the NHC group had a significant increase in the recruitment of CD68^+^ pan‐macrophages compared to the 100‐Pa HA hydrogel (*p* < 0.05) and 250‐Pa HA hydrogel (*p* < 0.01) controls (**Figure** **3**A–C), consistent with the improved cell migration observed in NHC in vitro (Figure 2C,D). Additionally, both CD38^+^ M1‐like macrophages (*p* < 0.001) and CD163^+^ M2‐like macrophages (*p* < 0.05) were recruited at higher levels in the NHC group (Figure 3B,D,E). On Day 14, similar trends were observed for CD68^+^ and CD38^+^ cells, but the CD163^+^ M2‐like macrophage prevalence in the NHC group was notably higher compared to the 100‐Pa HA hydrogel (*p* < 0.01) and 250‐Pa HA hydrogel (*p* < 0.001) controls (Figure 3C–E). On Day 56, consistent with the H&E histochemistry results, the immunofluorescence images showed no significant differences in the number of infiltrated CD68^+^ pan‐macrophages among the test groups (Figure 3C; Figure S6A–C, Supporting Information), suggesting that the recruitment of macrophages had halted. Furthermore, a significant decrease in CD38^+^ M1‐like macrophage density in the NHC group was observed, accompanied by an increase in CD163^+^ M2‐like macrophages on Day 56 in the NHC group. This skewed polarization of M2‐like and M1‐like macrophage phenotypes drastically altered the M2/M1 ratio (Figure 3F), which is a hallmark of pro‐regeneration response versus pro‐fibrotic inflammation.^[^ ^20^ ^]^ On the contrary, the HA hydrogel controls did not show such skewed polarization of macrophage phenotypes. These results suggest that NHC engenders early macrophage infiltration and pro‐regenerative M2‐like polarization (Figures 1C and 3E).

The pattern of early regenerative inflammation induced by NHC was confirmed by quantitative gene expression analysis. Pan‐macrophage marker CD68 was up‐regulated more in the NHCs on Day 7, 14, and 56 compared to the HA controls, indicating a continuous bio‐stimulatory effect for macrophage recruitment (Figure 3G). Further, M1‐specific genes NOS1 and CD38, showed early elevation by 5‐fold in the NHC matrix compared with HA controls on Day 7, with a decrease thereafter (Figure 3G). By contrast, M2‐specific genes Arg1 and CD163 genes were expressed early and persisted through the intermediate time points in the NHC matrix (Figure 3G).

**Figure 4:**
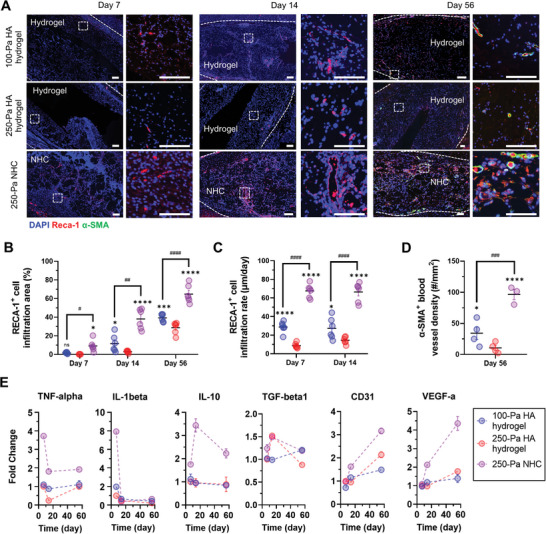
Improved host endothelial cell ingrowth followed by improved angiogenesis inside NHCs after subcutaneous injections. A) Host endothelial cell and smooth muscle in‐growth in the injected 100‐ and 250‐Pa HA hydrogels and the 250‐Pa NHC on Days 7, 14, and 56. Endothelial cells were stained with RECA‐1 (red), smooth muscles were stained with α‐SMA (green), and all infiltrated cell nuclei were stained with DAPI (blue). Scale bars = 200 µm for tile scans (left) and 50 µm for Z‐stack images (right). (B, C) Quantitative analysis of the endothelial cell infiltration area B) and the infiltration rate C) inside the hydrogels and the composite (*n =* 5). D) Quantitative Analysis of α‐SMA^+^ pericytes forming inside the hydrogels and the composite (*n =* 3). E) The gene expression level of tested genes for inflammation (TNF‐α and IL‐1β), anti‐inflammation (TGF‐β1 and IL‐10), and angiogenesis (CD31 and VEGF‐a). Expression is normalized to Day 7 250‐Pa HA. Samples were pooled together and then tested in triplicate (*n =* 3). Statistical significance was calculated by two‐way ANOVA with Dunnett's post hoc test. Hash key (#) denotes the comparison between the 100‐Pa HA hydrogel and the 250‐Pa NHC. Asterisk (*) indicates the comparison between the 250‐Pa HA control and the 100‐Pa HA or the 250‐Pa NHC. ns, *p* > 0.05, ^#^ or *p < 0.05, ^##^ or **p < 0.01, ^###^ or ***p < 0.001, ^####^ or ****p < 0.0001. Data are presented as means ± SEM.

To understand if the programmed macrophage polarization altered the local inflammatory behavior at the gene expression level, a few pro‐inflammatory genes (TNF‐α and IL‐1β), and pro‐regenerative genes (IL‐10 and TGF‐β1) were measured using qPCR. The qPCR results revealed that these two pro‐inflammatory genes were up‐regulated (3.7‐fold and 7.9‐fold, respectively) at the Day 7 timepoint, and then down‐regulated to lower levels (1.7‐fold and 0.4‐fold, respectively) in the NHC matrix (**Figure** **4**E). Meanwhile, two anti‐inflammatory genes, IL‐10 and TGF‐β1, were selected to measure the pro‐regenerative condition. Like the observations in macrophage polarization, IL‐10 was up‐regulated in the NHC matrix, indicating the onset of pro‐regenerative response, whereas measurements of TGF‐β1 did not show significant differences among the three groups (Figure 4E).

### 2.5 Biostimulatory NHC‐Mediated Endothelial Infiltration and Angiogenesis

A striking aspect of the remodeling responses evoked by NHC is the abundant vascularization throughout the injected area. The NHC group demonstrated the largest area of endothelial cell infiltration [250‐Pa NHC: 9.2 ± 6.1%, 100‐Pa HA hydrogel: 1.7 ± 0.6% (*p* < 0.05), and 250‐Pa HA hydrogel: 0.2 ± 0.1% (*p* < 0.05); Figure 4A,B] as well as a markedly higher rate of endothelial cell infiltration, 67.5 ± 7.2 µm per day when compared to the 100‐Pa HA and 250‐Pa HA hydrogel groups on day 7 (*p* < 0.0001; Figure 4A,C). On Day 14, a similar trend was observed in terms of the endothelial cell infiltration rate and area, with the NHC group remaining at a high level of 66.5 ± 10.4 µm per day (*p* < 0.0001 compared to HA hydrogel controls) and the endothelial cells starting to spread broadly within the NHC (Figure 4A). Importantly, these infiltrating cells formed functional blood vessels, as confirmed by α‐SMA^+^ staining, suggesting neointima formation around the endothelial tubes (Figure 4A,D). The development of vasculature in NHC was accompanied by enhanced expression of pro‐angiogenic markers CD31 and VEGF in NHC (Figure 4E). Given that M2 macrophages are known to secrete pro‐angiogenic cytokines, we examined the correlation between macrophage infiltration and vascularization. CD163^+^ M2‐like macrophages were localized in close proximity to RECA‐1^+^ endothelial cell clusters throughout the early remodeling period,^[^ ^21^ ^]^ suggesting that the early vasculature formation may be supported by the pro‐regenerative macrophages (Figure S6D, Supporting Information). This finding further supports the potential of the NHC in promoting tissue regeneration through the promotion of angiogenic response and the skewed polarization of macrophages (Figure 1C).

### 2.6 Enhanced Adipocyte Formation Following the Inflammation‐Induced Angiogenesis

**Figure 5:**
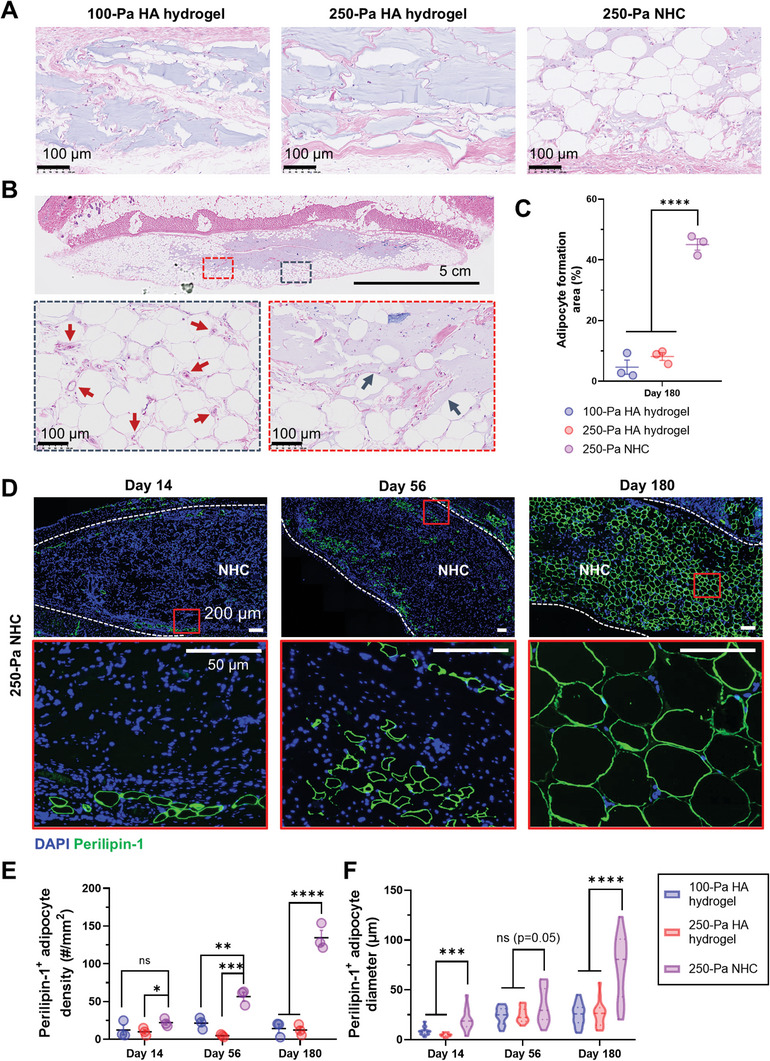
NHC‐mediated adipocyte formation and adipose tissue remodeling. A) H&E staining images of the 100‐ and 250‐Pa HA and 250‐Pa NHC on Day 180. Scale bar = 100 µm. B) Zoomed‐in and zoomed‐ out H&E staining images of the 250‐Pa NHC on Day 180. Scale bar = 100 µm for zoomed‐in images. Red arrows indicate blood vessels and black arrows indicate the interface between the NHC and adipocytes. C) Quantitative analysis of the area of adipocyte formation based on the H&E staining images collected on Day 180 (*n =* 3). D) Immunocytochemistry images for the 250‐Pa NHC on Days 14, 56, and 180. Adipocytes were stained with Perilipin‐1 (green), and all infiltrated cell nuclei were stained with DAPI (blue). Scale bars = 200 µm for tile‐scans (top) and 50 µm for z‐stack images (bottom). E) Quantitative analysis of the density (*n =* 3) and F) the diameter F) of adipocytes inside the NHC and HA hydrogels (*n =* 30, 10 random adipocytes measured for each immunostaining image). Statistical significance was calculated by one‐way ANOVA with Dunnett's post hoc test to compare between groups. ***p < 0.001, ****p < 0.0001. Data are presented as means ± SEM.

Soft tissue volume restoration by NHC is biphasic, with early vascularization provided by the matrix alone and longer‐term restoration driven by de novo vascularized adipose tissue formation as the NHC is remodeled and degraded. The new NHC formulations developed here show a striking capacity for adipose tissue formation without the need for exogenous inductive factors. Six months following NHC injection, adipocytes were widespread inside the NHC matrix, occupying ≈45% of the total area within the NHC (**Figure** **5**A–C). The adipocytes were interdigitated with remaining NHC microgels adjacent to developing blood vessels (Figures 1C and 5B). We next performed Perilipin‐1 immunostaining – a characteristic marker of lipid droplets to mark adipocytes.^[^ ^22^ ^]^ Confocal images revealed that starting on Day 14, newly formed adipocytes started to appear inside the NHC close to the periphery, with the number and size of those adipocytes increasing through Day 56 to 180 (Figure 5D,E). In contrast, adipocytes were rarely observed inside HA hydrogel controls at all three time points (Figures S7 and S8, Supporting Information). Quantitative analysis revealed that on Day 14, the density of Perilipin‐1^+^ adipocytes within the NHC was approximately two‐fold higher than the 250‐Pa HA hydrogel control group (*p* < 0.05) but was not significantly different from that in 100‐Pa HA hydrogel control (*p* > 0.05; Figure 5E; Figures S5 and S6, Supporting Information). However, the density of adipocytes continued to increase in NHC with time, and by Day 180, the density of adipocytes reached 134.5 ± 14.0 cells mm^−2^, five‐fold higher than either hydrogel control (*p* < 0.0001; Figure 5E; Figures S5 and S6, Supporting Information). Not only were there more adipocytes, but they were significantly larger with mature lipid droplets in the NHC at Day 180 (Figure 5D,F). These findings suggest that NHC not only supports the formation of adipocytes but also provides a favorable microenvironment for their growth and maturation.

### 2.7 Programmed Inflammation Induces Progenitor Cell Infiltration to Drive Adipogenesis

We next examined the mechanism of abundant vascularized adipose tissue formation in NHC. In the absence of exogenous cues, one possibility is that the regenerative inflammation engendered by NHC might itself be adipogenic. First, we co‐stained the adipose tissue marker adiponectin (Acrp‐30) with Perilipin‐1 inside the NHCs. Results showed that Acrp‐30 expression was also elevated inside NHCs compared to HA hydrogel controls on Days 14 and 56 (Figure S7A, Supporting Information). Interestingly, Acrp‐30 can be expressed on endothelial cells as well as adipocytes, and some vascular structures were observed very close to adipocytes in the remodeling NHC (Figure S7A, Supporting Information). These observations led us to hypothesize that adipocyte formation may be linked to the infiltration and formation of blood vessels (Figure 1C).

**Figure 6:**
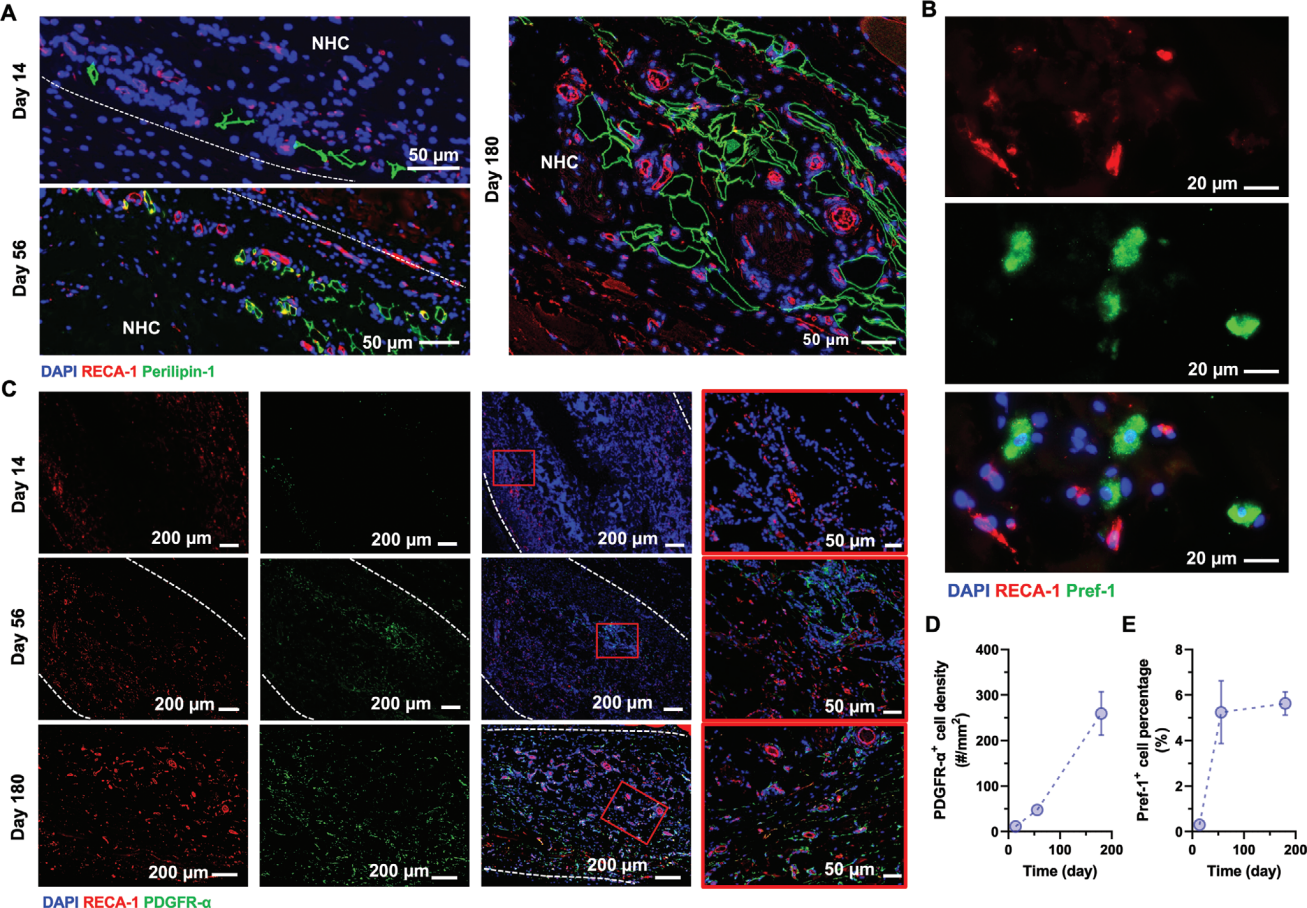
Close correlation of adipocyte formation to perivascular progenitor infiltration. A–C) Immunocytochemistry images for the 250‐Pa NHC group on Days 14, 56, and 180, show the close localization of A) adipocytes with blood vessels, B) pre‐adipocytes, and C) PDGFRa+ perivascular cells. D) Quantitative analysis of the density of PDGFRa^+^ perivascular cells on Days 14, 56, and 180 in the 250‐Pa NHC (*n =* 3). E) Quantitative analysis of the density of pre‐adipocytes on Days 14, 56, and 180 in the 250‐Pa NHC (*n =* 3). Statistical significance was calculated by one‐way ANOVA with Dunnett's post hoc test to compare between groups. ***p < 0.001, ****p < 0.0001. Data are presented as means ± SEM.

To further investigate this correlation, immunofluorescence of Reca‐1^+^ endothelial cells and Perilipin‐1^+^ adipocytes was performed. We observed a close localization of endothelial cells and adipocytes on Days 14, 56, and 180 inside the 250‐Pa NHC (**Figure** **6**A). Then, a co‐staining of Pref‐1^+^ pre‐adipocytes and Reca‐1^+^ endothelial cells was performed to understand the correlation between vasculature formation and differentiation or the origin of adipocytes. In confocal imaging, Pref‐1^+^ pre‐adipocytes were frequently found localized near endothelial cells. The quantitation revealed that on Day 14, almost no Pref‐1^+^ cells were observed inside NHC. However, on Day 56, ≈5% of cells started to express the pre‐adipocyte marker, and this percentage remained consistent until Day 180 (Figure 6B,E; Figure S9, Supporting Information). A number of recent studies have identified perivascular cell populations, particularly PDGFRα^+^ cells, as adipocyte stem cells capable of differentiating into preadipocytes and, finally, mature adipocytes.^[^ ^23^ ^]^ Strikingly, immunofluorescence images showed that the number of PDGFRα+ cells continued to increase from Day 14 to 180 in NHC, and they were closely localized near the endothelial cells (Figure 6C,D). These results, coupled with the kinetics of tissue remodeling, suggest a self‐sufficient mechanism for NHC to generate adipose tissue in vivo. Specifically, NHC recruits and polarizes macrophages in a pro‐regenerative direction, leading to angiogenesis and recruitment of adipocyte precursors in the perivascular space.

## 3 Discussion

In this study, we have developed a new granular hydrogel composite that incorporates collagen nanofibers. This composite is characterized by its porous structures and mechanical enhancement features. The collagen nanofibers, acting as the structural reinforcement component in NHC, increase the shear storage modulus, affording the structural integrity of the injected microgels and maintaining the pore size of the hydrogel phase following subcutaneous injection, which is critical to host cell infiltration. Our data clearly demonstrated the sequential events that point to the roles of the collagen nanofibers in the NHC in terms of sustaining the recruitment of host immune cells, mediating their migration and spreading inside the matrix, and gradually generating and maintaining a diverse subset of macrophages with an increasing amount of pro‐regenerative macrophages, likely due to the mechanical and paracrine conditioning inside the soft hydrogel matrix. These actions are important for amplifying the proangiogenic effect and increasing the amounts of endothelial cells and pericytes infiltrating into the NHC matrix. The sustained recruitment of PDGFRα+ perivascular progenitor cells, which then differentiate into adipocytes inside the NHC, is essential to the observed adipogenesis outcome. In contrast to our previous investigations using PCL‐based NHC, which primarily focused on the angiogenic behavior steered by the NHC matrix, the current study pivots on how the NHC matrix could orchestrate the inflammatory responses toward regenerative soft tissue formation.

Inflammation is a crucial tissue response to injury, infection, and tissue loss to contain damage and promote healing. Depending on the local tissue microenvironment and the nature of exogenous stimuli, inflammation can be acute and controlled or prolonged with attendant fibrosis and scarring. Recent studies indicate that inflammatory responses may be programmed by implanted biomaterials to optimize pro‐regenerative responses in different tissues.^[^ ^10^, ^24^ ^]^ Macrophages are key early regulators of these responses; by altering macrophage polarization, biomaterials can incite fibrosis or pro‐regenerative tissue restoration. In the present work, by introducing a biostimulatory collagen nanofiber‐HA hydrogel composite matrix subcutaneously, we were able to create a sustained and mild inflammatory microenvironment with continuous host macrophage recruitment. Instead of deteriorating toward chronic inflammation and fibrotic tissue formation, this NHC matrix promoted a pro‐regenerative remodeling environment. Specifically, NHC induced higher CD163^+^ macrophage polarization compared to the HA controls with matching pore size and stiffness, respectively. Following the macrophage polarization, reparative genes such as IL‐10 and VEGF‐α were upregulated in NHC, driving higher levels of angiogenesis and soft tissue over six months of implantation and remodeling. Importantly, these outcomes are achieved without the need for exogenous growth factors, easing the path to manufacturing and clinical translation. Building on our prior work with PCL nanofiber‐based NHC, the current study suggests that the capacity program regenerative inflammation inheres to the physical and structural properties of NHC and is not tied to a single set of chemical constituents.

In addition to the elevated angiogenic behaviors and adipose tissue formation, this study also underscores the correlation between NHC‐mediated angiogenesis and soft tissue formation. We observed that newly formed pre‐adipocytes and mature adipocytes were closely localized with endothelial cells, forming a vascularized adipose tissue within the collagen NHC matrix. Considering that abundant host cells were recruited to the NHC matrix by the stimulatory collagen nanofibers, we then hypothesize that a number of perivascular progenitor cells were also recruited. The immunostaining images reveal a robust spatiotemporal correlation between the infiltration of PDGFRα^+^ perivascular progenitor cells and adipose tissue formation within the remodeling matrix. This finding aligns with previous studies where PDGFRα^+^ progenitor cells are considered a major cell source differentiating into white adipocytes. This suggestive finding can be explored in future lineage tracing studies such as the PDGFRα‐CreER; mT/mG mouse model to elucidate the precise origin of growing adipocytes and to reveal the mechanism of adipogenesis and angiogenesis within injected NHCs.

In summary, we have developed a granular collagen nanofiber‐reinforced hydrogel composite that functions as a soft tissue substitute, mimicking the mechanical properties and microarchitecture of the native tissue. The porous structure of NHC, with its reinforcing collagen fibers, facilitates host cell infiltration. The NHC further programs these infiltrating cells in a pro‐regenerative direction, with specific polarization of macrophages toward an M2‐like phenotype. These cells, in turn, promote angiogenesis and recruit perivascular PDGFRα^+^ adipocyte precursors, ultimately resulting in vascularized adipose tissue formation. The capacity of these nanomaterials to program regenerative inflammation as a function of their physicochemical properties alone and in the absence of exogenous differentiation cues heralds an important new strategy for soft tissue restoration.

## 4 Experimental Section

### Materials

Sodium hyaluronate (MW 1.5 MDa) was purchased from LifeCore. Bovine type I collagen solution was purchased from Advanced Biomatrix. HUVECs and vascular endothelial cell culture medium were purchased from Lonza. All other chemical reagents were purchased from Sigma–Aldrich. All other cell culture reagents and supplements were obtained from Invitrogen.

### Preparation of Collagen Nanofibers

Bovine collagen solution was first lyophilized overnight to obtain collagen powders, and then type I collagen solution (8 w/v%) was prepared in HFIP at room temperature for ≈6 h to make a viscous cloudy electrospinning solution. The electrospinning was performed with the following parameters: 5 mL h^−1^ of the flow rate; 20–25 kV of the voltage applied to the 22‐G metallic needle; 12.5 cm of the collecting distance; 900 rpm of the rotation rate of the metallic collector. By using carbodiimide chemistry, fibers were immersed in ethanol solution (95 v/v%) containing 50 mm 1‐ethyl‐3‐(3‐dimethyl aminopropyl) carbodiimide (EDC) and 20 mm N‐hydroxysuccinimide (NHS) for 24 h. After the cross–linking, fibers were washed in 0.75 w/v% glycine solution three times for 5 min each time to remove the excessive reagents and quench the activated fiber surface. The collagen fibers were then broken down into fragments using cryomilling (Freezer/Mill 6770, SPEX SamplePrep). The fragments were filtered through different cell strainers (40 and 100 µm) to reach a relatively uniform fiber length.

### Preparation and Characterization of HA Hydrogels and NHCs

HA was dissolved in distilled water at a stock concentration of 25 mg mL^−1^. DVS concentration was calculated as the ratio to the hydroxyl groups in HA (such as 0.5×, 1×, and 2×). The stock HA solution was diluted to 2 w/v% using distilled water and sodium hydroxide to get four different pH values (12.4, 12.7, 13.0, and 13.3), with other parameters set to be the same (2 w/v% HA, 37 °C, 2 h reaction time). The reaction time or gelation kinetics were performed by preparing multiple samples and measuring the mechanical properties at various time points (0.5, 1, 2, 3, 4, 8, and 16 h) to get a time point where the stiffness reaches a plateau. The cross–linking of the NHCs followed the same conditions as the HA hydrogels; different fiber densities (0, 1, and 3 w/v%) were added to the mixed precursors to test the gelation kinetics of the NHCs. After the gelation of hydrogels and NHCs, dialysis was performed using dialysis membranes (6 – 8 KDa MWCO, Spectrum Labs) against pH 7.4 phosphate buffer for 48 h to remove the unreacted DVS, to balance the pH and to swell the samples for further studies. The mechanical properties were measured again after the swelling. Microgels were then generated with stainless steel wire cloth discs to reach a gel particle size of ≈150 µm, as we previously reported.^[^ ^25^ ^]^

### Sterilization of Hydrogels and Composites

Autoclaving was performed to sterilize the hydrogels and NHCs after gelation. Briefly, the gels were placed at the autoclave cycle at 118 °C with a 5 min sterilization step. The total sterilization cycle would take 30 min to complete. After the sterilization, the mechanical properties of each gel were measured again using rheological tests.

### Mechanical Properties of Hydrogels and NHCs

The rheological characterization of hydrogels and NHCs was performed as described previously. An AR2 Rheometer (TA Instruments) was used to measure various shear mechanical properties of hydrogels and NHCs using a parallel plate with a diameter of 8 mm with 0.5 mm gap at 25 °C. A linear viscoelastic region was measured for the gels by a strain sweep with an increasing shear strain amplitude at a set frequency (1 Hz). The three main properties were storage modulus (*G*’), loss modulus (*G*’’), and tanδ (*G*’’/*G*’). The injection forces of bulk gels and microgels of HA and NHC were measured by extruding a 1‐mL sample through a 27‐G NIPRO needle in a 1 mL BD syringe using an Instron load frame (34SC‐05, Norwood, MA).

### Cell Culture

Human umbilical vein endothelial cells were purchased from Lonza and were cultured in EGM (Lonza) and incubated at 37 °C under 5% CO_2_. Human adipose‐derived stem cells (hADSCs) were maintained in Dulbecco's Modified Eagle Medium media with 10% fetal bovine serum and incubated at 37 °C under 5% CO_2_ and used before passage 3 in this study. Cell spheroids with a uniform size of 200 µm were fabricated using an agarose hydrogel microwell. For the 3D culture of hADSC spheroids, the plate was coated with cross–linked hydrogels or NHCs and centrifuged at 300 ×g for 5 min. Then, hADSC spheroids mixed with hydrogels or NHCs were seeded on top of pre‐formed gels and cultured for 7 days.

### In vitro Assessments

Cell viability was analyzed using a Live/dead cell viability kit (Sigma–Aldrich). For each cell type, cells were seeded (50 µL, 5 × 10^6^ cells mL^−1^) in a 96‐well plate coated with HA hydrogels or NHC containing 150 µL medium and cultured for 1, 4, and 7 days. Cell adhesion assay was performed similarly by seeding cells in pre‐coated wells, and after 4, 24, and 48 h, the cell numbers in the media suspension were measured to count the percentage of the unadhered cells. Cell migration and spreading of hADSCs in 2D and 3D cultures were examined by immunostaining. The cells were fixed with 4 w/v% paraformaldehyde for 10 min. And then stained with Phalloidin 568. Cell nuclei were counterstained with 4′, 6‐diamidino‐2‐phenylindole dihydrochloride (DAPI, Molecular Probes). All the images were acquired by LSM 780 Confocal Microscope.

### Subcutaneous Injection in Rats

All animal procedures were performed under an animal protocol approved by the Johns Hopkins Institutional Animal Care and Use Committee (Protocol no. RA24E63; Development of biocompatible nanofiber‐hydrogel composites for stem cell delivery and soft tissue regeneration). To investigate the NHC‐mediated macrophage polarization, angiogenesis, and adipogenesis in vivo, NHCs and hydrogels were injected into the subcutaneous space of CD IGS (Sprague Dawley) rats (6–8 weeks old, female, 150–200 g). Three replicates were performed for each formulation at a volume of 200 µL per injection. The rats were sacrificed at 7, 14, 56, and 180 days after injection. The explant sizes were measured by calipers to get a rough geometry of the retained volume The injection explants were then immediately reserved for immunohistochemistry in 4 w/v% paraformaldehyde (PFA) for 3 days after sacrificing the animals. The explants were then embedded in paraffin after a series of dehydration, followed by the sectioning to get slices within 10 µm.

### Histology Assessment

The tissue slices embedded in paraffin slices were deparaffinized and then stained with hematoxylin and eosin (H&E) staining and Masson's trichrome staining for histological analysis. For quantitative analysis, three slices were stained for each injection.

### Immunofluorescence Imaging

The tissue slices embedded in paraffin slices were deparaffinized and rehydrated to activate the surface for immunostaining. Briefly, explants were immersed in xylene, 100%, 95%, and 70% ethanol in series for 10 min each and then placed in IHC buffer inside a boiling pot for 20 min to activate the tissue surface. Sections then were permeabilized with 0.5% Triton X‐100 solution and blocked by 4% donkey serum in PBS for 2 h. The samples were then incubated with primary antibodies (Table S1, Supporting Information) overnight at 4 °C. Cy3 and Cy5 affinity secondary antibodies (Jackson ImmunoResearch Laboratories, Table S2, Supporting Information) were applied to the sections at room temperature for 2 h. Sections were then washed three times with PBS, and counterstained with DAPI for 15 min. Six random fields of each specimen were imaged by ZEISS LSM 780 Confocal Microscope for quantitative analysis.

### RNA Isolation & RT‐qPCR Analysis

As previously described, RNA was isolated from samples snap‐frozen in liquid nitrogen immediately following excision, using the TRIzol protocol (ThermoFisher) and a power homogenizer (Polytron/VWR). Briefly, RNA was precipitated, washed twice with 75% ethanol, dried, and resolubilized in RNAse‐free water. Given thorough sample homogenization, displayed gene‐expression signatures are representative of host response on and/or infiltrating into implanted materials. Nucleic acid quality and concentration were measured using a NanoDrop 2000 spectrophotometer (ThermoFisher).

All samples were normalized by loading 1 µg total RNA in for reverse transcription (RT) of RNA into separate 0.2 mL PCR tubes (Fisherbrand). DEPC‐water was used to equilibrate each sample volume to 12.2 µL, and then 2 µL of 10× RT buffer, 0.8 µL of dNTPs, 1 µL of DNAse, and 1 µL of RNAse inhibitor was added to each sample (High‐Capacity cDNA Reverse Transcription Kit, Applied Biosystems; DNAse, Promega; RNase Inhibitor, ThermoFisher). A DNAse cycle (1 h at 37 °C, 10 min at 75 °C, infinite hold at 4 °C) was run to remove any potentially contaminating genomic DNA. DNAse‐free samples had 2 µL of 10× RT random primers and 1 µL of reverse transcriptase enzyme added and were placed back onto a ThermoCycler (Applied Biosystems) to reverse transcribe RNA into cDNA. The resultant cDNA was then subjected to expansion and analysis by qPCR using the Power SYBR Green protocol (Applied Biosystems). Samples were incubated for 2 min at 50 °C then 10 min at 95 °C followed by 40 cycles of 95 °C for 15 sec and 60 °C for 60 s in a QuantStudio 5 Real‐Time PCR System. Results were analyzed by the comparative CT (∆∆CT) method and are presented as RNA levels relative to controls, as indicated.

The following primers (forward and reverse, respectively) were used for CD68: 5′‐GCCACAGTACAGTCTACCTTA‐3′ and 5′‐AGAGATGAATTCTGCGCTGA‐3′; for Nos2: 5′‐AGGTTGGAGGCCTTGTGTC‐3′ and 5′‐GCTTCAGAATGGGGAGCTG‐3′; for CD86: 5′‐CGTCAAGACATGTGTAACCTGC‐3′ and 5′‐AAGCTTGCCTCTTCACAGGA‐3′; for Arg1: 5′‐CCGCAGCATTAAGGAAAGC‐3′ and 5′‐CCCGTGGTCTCTCACATTG‐3′; for CD163: 5′‐TCAGCGTCTCTGCTGTCACT‐3′ and 5′‐CGGCCAGTCTCAGTTCCTT‐3′; for CD38: 5′‐AGCACCTTTGGAAGTGTGGA‐3′ and 5′‐AGGGGCTCGAACATGCATTA‐3′; for TNF‐α: 5′‐CACGCTCTTCTGTCTACTGAACTTC‐3′ and 5′‐GAGTGTGAGGGTCTGGGCCATG‐3′; for IL1β: 5′‐TGGCAACTGTCCCTGAACTC‐3′ and 5′‐CCCAAGTCAAGGGCTTGGAA‐3′; for TGF‐β1: 5′‐CCTGGAAAGGGCTCAACAC‐3′ and 5′‐TGCCGTACACAGCAGTTCTT‐3′; for IL10: 5′‐AGTGGAGCAGGTGAAGAATGA‐3′ and 5′‐TCATGGCCTTGTAGACACCTT‐3′;; for CD31: 5′‐AGTGTGGAAACCAACAGCCA‐3′ and 5′‐GCTCAAGGGAGGACACTTCC‐3′; for VEGF‐a: 5′‐CCAAAGCCAGCACATAGGAG‐3′ and 5′‐ TGGCTTTGTTCTATCTTTCTTTGGT ‐3′; and for housekeeping gene HPRT: 5′‐CCTCCTCAGACCGCTTTTCC‐3′ and 5′‐AGGTCATAACCTGGTTCATCATCA‐3′.

### AI Disclosure

During the editing of this manuscript, ChatGPT was utilized to enhance the clarity and coherence of our writing, including improving grammar and suggesting alternative phrasing to better convey our ideas. It was important to note that while AI contributed to the editing process, the final content and interpretations remain the sole responsibility of the authors. It was also ensured that the use of AI adheres to ethical standards and maintains the integrity of the research.

### Statistical Analysis

Statistical analysis Data are shown as means ± SEM. Data were analyzed using GraphPad Prism software (GraphPad Software Inc.) by Student's t‐test (unpaired and two‐tailed), one‐ or two‐way ANOVA (analysis of variance), followed by Dunnett's, Tukey's, or Bonferroni's post hoc test as needed. The values were considered significantly different at *p* < 0.05.

## Conflict of Interest

H.Q.M. and S.K.R. are co‐inventors on three issued US patents related to this work filed by Johns Hopkins University (U.S. Patent No. 10463768 B2, granted on 5 November 2019; U.S. Patent Application No. 11684700 B2, granted on 27 June 2023; U.S. Patent Application 11707553 B2, granted on 25 July 2023). The authors declare no other competing interests.

## Author Contributions

H.Q.M., S.K.R. conceived of this study. S.K.R., H.Q.M., J.K., and Y.‐H.Y. designed the study. J.K., Z.C.Y., J.L.S., J.C., and C.S. performed the experiments. J.K., J.L.S, Y.H.Y., J.C, H.F., C.S., C.Z., K.K., L.C., J.P., K.D., Y.Z., X.L., J.C.D., S.K.R., and H.Q.M. participated in data analysis and interpretation. The manuscript was written by J.K., H.Q.M., and S.K.R. with inputs from all the other authors. S.K.R. and H.Q.M. secured the funding for this study.

## Supporting information



Supporting Information

## Data Availability

The data that support the findings of this study are available from the corresponding author upon reasonable request.
