# Supplementary material for: Granular Nanofiber‐Hydrogel Composite‐Programmed Regenerative Inflammation and Adipose Tissue Formation
Source: Adv Healthc Mater. 2024 Nov 24;14(3):2403094. doi: 10.1002/adhm.202403094 (PMC11773126; doi:10.1002/adhm.202403094)
Supplement: Supplementary file 1 — Supporting Information [file ADHM-14-0-s001.docx]

***Supporting Information***

**Granular Nanofiber-Hydrogel Composite-Programmed Regenerative Inflammation and Adipose Tissue Formation**

Jiayuan Kong^a,b,c^, Zhi-Cheng Yao^a,b,c^, Jessica L. Stelzel^b,c,d^, Yueh-Hsun Yang^b,e^, Jeffrey Chen^b,c,d^, Hexiang Feng^b,c,d^, Collin Schmidt^a,b,c^, Chi Zhang^a,b,c^, Kedar Krishnan^b,c,d^, Long Chen^f^, Jingwen Pan^d^, Kailei Ding^a,b,c^, Yining Zhu^b,c,d^, Xiaowei Li^g^, Joshua C. Doloff^a,b,c,d^, Hai-Quan Mao^a,b,c,d,1^ and Sashank K. Reddy^b,c,d,e,1^

^a^Department of Materials Science and Engineering, Johns Hopkins University, Baltimore, MD 21218, USA

^b^Translational Tissue Engineering Centre, Johns Hopkins University School of Medicine, Baltimore, MD 21213, USA

^c^Institute for NanoBioTechnology, Johns Hopkins University, Baltimore, MD 21218, USA

^d^Department of Biomedical Engineering, Johns Hopkins University School of Medicine, Baltimore, MD 21205, USA

^e^Department of Plastic and Reconstructive Surgery, Johns Hopkins School of Medicine, Baltimore, MD 21287, USA

^f^Department of Orthopaedics, Guizhou Provincial People's Hospital, Guiyang, Guizhou, 550000, P. R. China

^g^Department of Surgery, Washington University School of Medicine, St. Louis, MO 63110, USA

^1^To whom correspondence should be addressed. E-mails: sreddy6@jhmi.edu (S. R.) and hmao@jhu.edu (H.-Q. Mao).

**List of Contents**

**Supplementary Figures:**

**Figure S1.** Characterization of collagen nanofibers.

**Figure S2.** Characterization and the performance of HA hydrogels and NHCs crosslinked by DVS.

**Figure S3.** Imaging analysis of the crosslinked collagen nanofibers in the NHC.

**Figure S4.** Enhanced cell adhesion and migration by including collagen nanofibers in the NHC matrix.

**Figure S5.** Prolonged in vivo retention of NHCs in rat subcutaneous model.

**Figure S6.** Effect of NHC on the programming of local inflammation.

**Figure S7.** NHC-mediated adipose tissue formation.

**Figure S8.** Tile-scan immunostaining images in parallel with H/E staining images for 100-Pa and 250-Pa HA hydrogel controls and 250-Pa NHCs on Days 14, 56, and 180.

**Figure S9.** Immunocytochemistry images for 250-Pa NHC on Days 14, 56, and 180.

**Supplementary Tables:**

**Table S1.** Primary antibodies used in this study.

**Table S2.** Secondary antibodies used in this study.

**
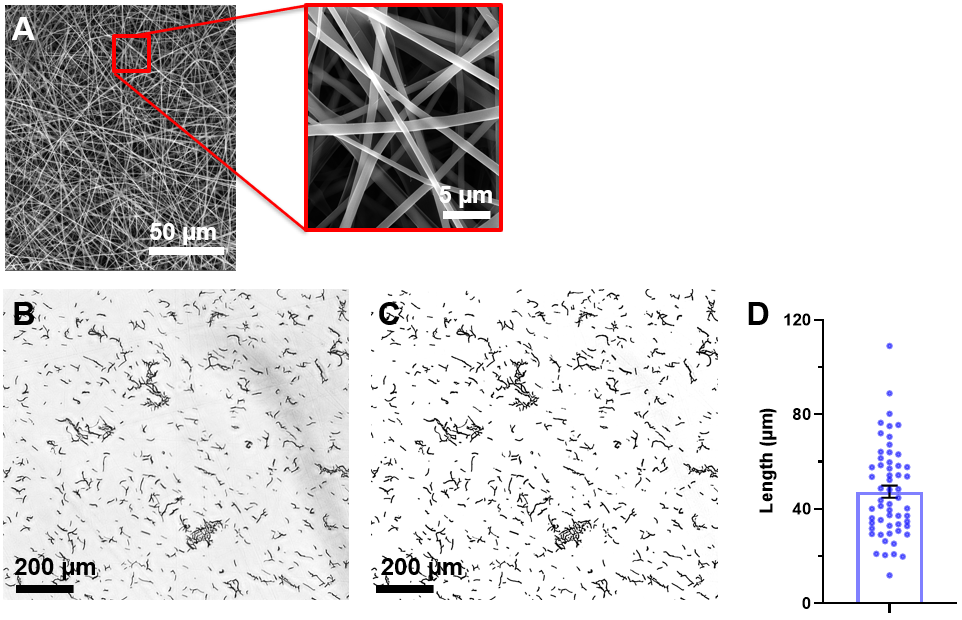
**

**Supplementary Figure S1. Characterization of collagen nanofibers.** (**A**) A representative SEM image of electrospun collagen nanofibers. (**B**) EDC/NHS-crosslinked nanofibers were dispersed in ethanol and imaged using a 40 × optical microscope. (**C–D**) The fiber images were processed to (C) 8-bit using ImageJ for (D) fiber length quantification (a minimum of 50 fibers were measured).


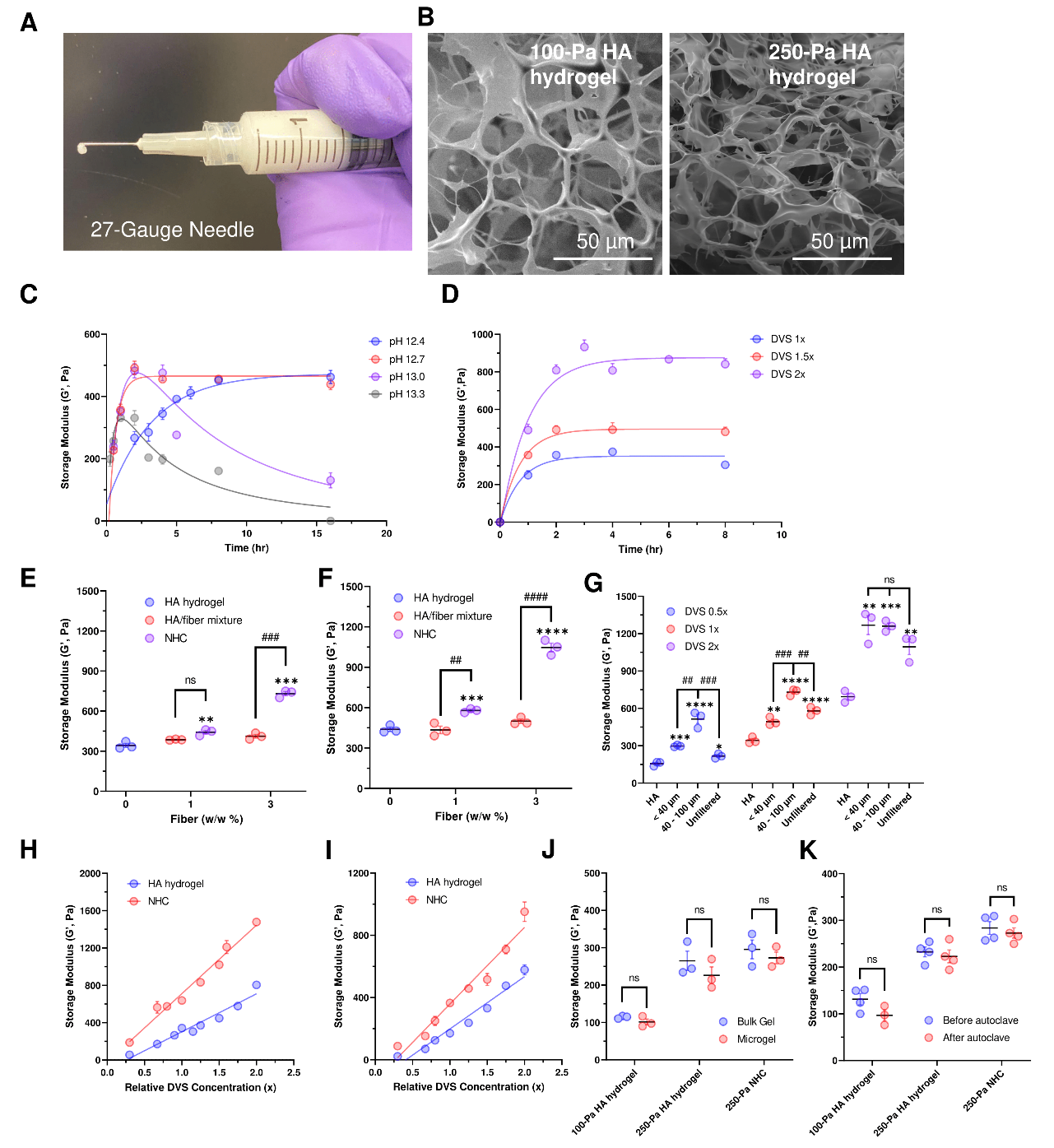


**Supplementary Figure S2. Characterization and the performance of HA hydrogels and NHCs crosslinked by DVS.** (**A**) An image to show the injectability of granular NHC through 27-G needle. (**B**) SEM images to visualize the pore structures of 100-Pa and 250-Pa HA hydrogel controls. (**C**) The crosslinking kinetics of HA hydrogels with 1.5× DVS at pH 12.4, 12.7, 13.0 and 13.3 prepared by different NaOH concentrations. (**D**) The crosslinking kinetics of HA hydrogels at pH 12.7 with different DVS concentrations (1×, 1.5×, and 2×). (**E**–**F**) HA hydrogels and NHCs with different DVS crosslinker concentrations of (E) 1.0× and (F) 1.5×. The reaction pH was maintained at pH 12.7 (n = 3 to 9). (**G**) HA hydrogels and NHCs with different fiber lengths screened by different sizes of cell strainers (40 µm, 100 µm, and unfiltered). (**H**–**I)** The storage modulus (G’) range of HA hydrogels and before (H) and after swelling (I). (**J-K)** before and after particulation (J), and before and after autoclaving (K).


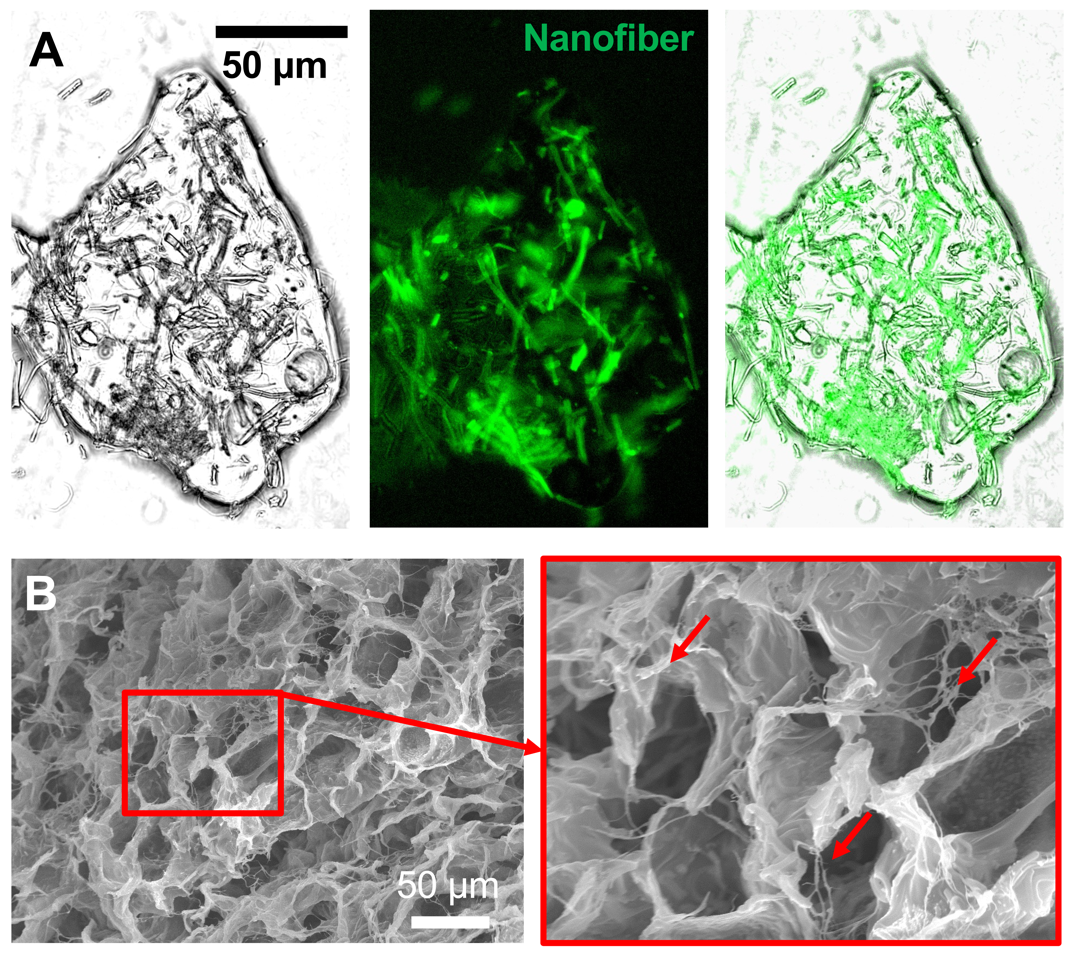


**Supplementary Figure S3. Imaging analysis of the crosslinked collagen nanofibers in the NHC.** (**A**) A representative phase contrast image of an NHC microparticle obtained using a 40× optical microscope (left panel), a fluorescence microscope image showing FITC-labeled collagen fiber fragments (middle), and an overlay of the two (right). (**B**) A representative SEM image of the NHC. Red arrows show the representative nanofiber fragments entangled within the HA hydrogel.


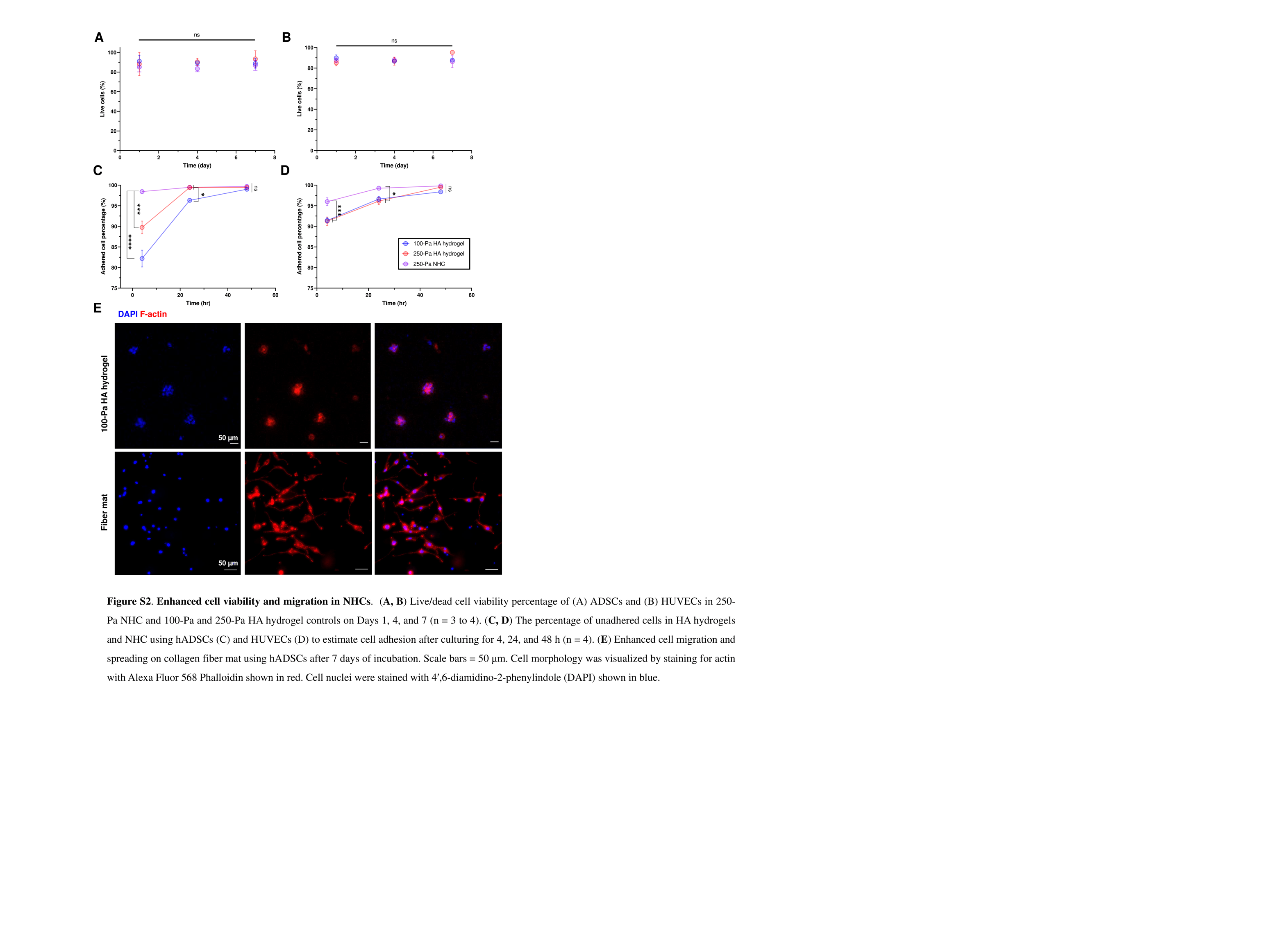


**Supplementary Figure S4**. **Enhanced cell adhesion and migration by including collagen nanofibers in NHC matrix**. (**A,** **B**) Live/dead cell viability percentage of (A) ADSCs and (B) HUVECs in 250-Pa NHC and 100-Pa and 250-Pa HA hydrogel controls on Days 1, 4, and 7 (n = 3 to 4). (**C,** **D**) The percentage of unadhered cells in HA hydrogels and NHC using hADSCs (C) and HUVECs (D) to estimate cell adhesion after culturing for 4, 24, and 48 h (n = 4). (**E**) Enhanced cell migration and spreading on collagen fiber mat using hADSCs after 7 days of incubation. Scale bars = 50 μm. Cell morphology was visualized by staining for actin with Alexa Fluor 568 Phalloidin shown in red. Cell nuclei were stained with 4′,6-diamidino-2-phenylindole (DAPI) shown in blue.

**
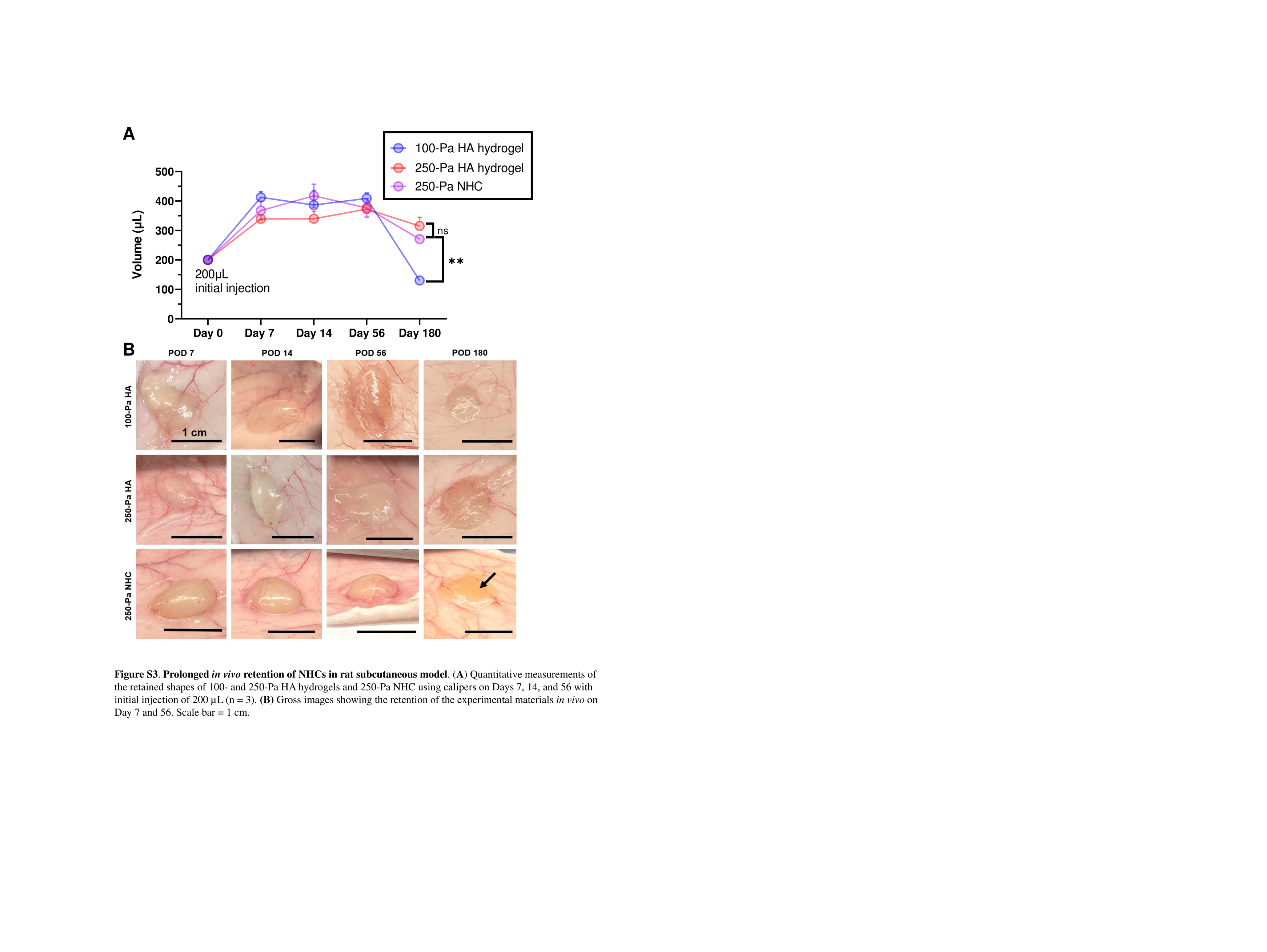
**

**Supplementary Figure S5**. **Prolonged *in vivo* retention of NHCs in rat subcutaneous model**. (**A**) Quantitative measurements of the retained shapes of 100- and 250-Pa HA hydrogels and 250-Pa NHC using calipers on Days 7, 14, and 56 with initial injection of 200 µL (n = 3). **(B)** Gross images showing the retention of the experimental materials *in vivo* on Days 7 and 56. Scale bar = 1 cm.

**
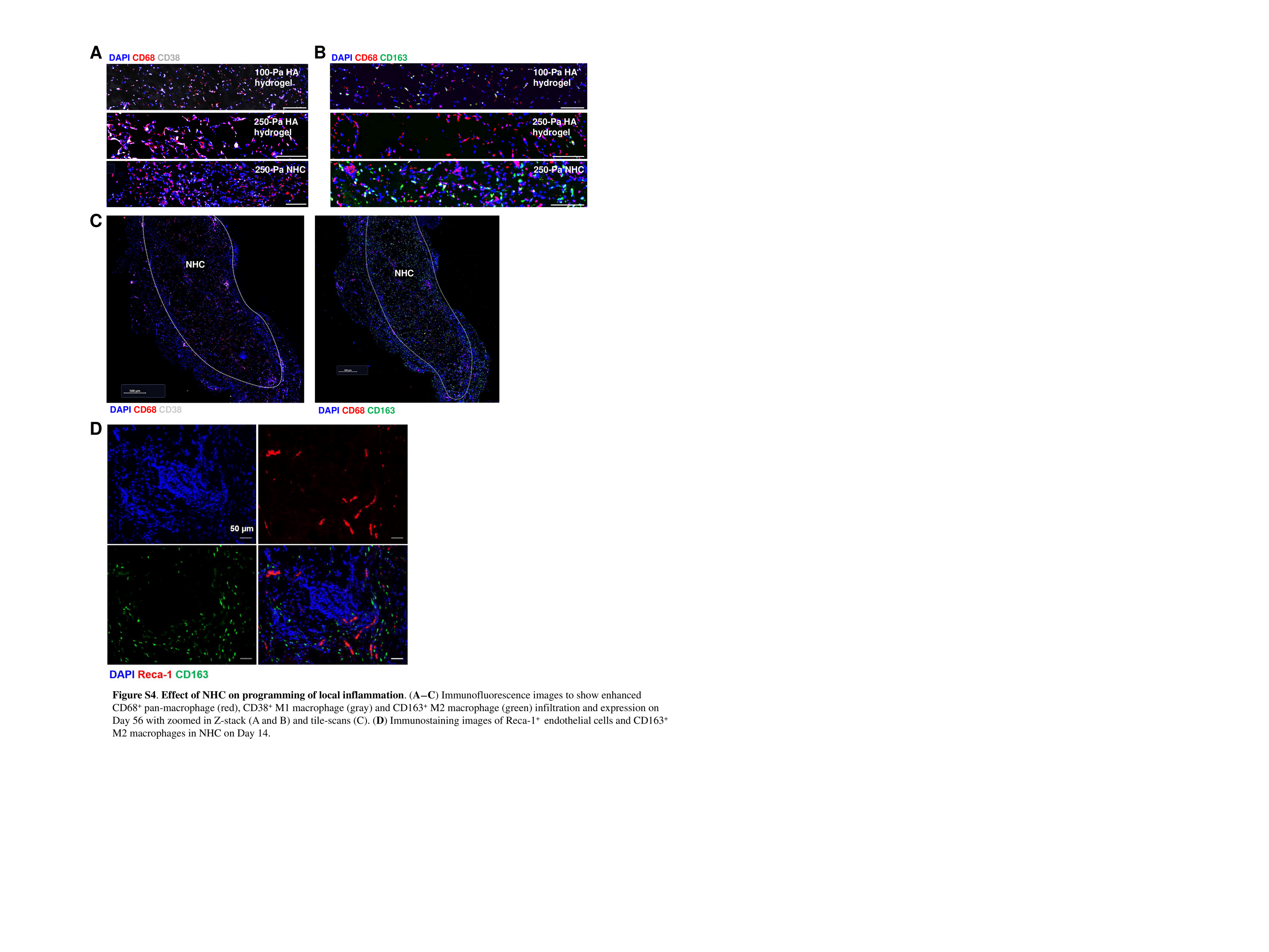
**

**Supplementary Figure S6**. **Effect of NHC on the programming of local inflammation**. (**A–C**) Immunofluorescence images to show enhanced CD68^+^ pan-macrophage (red), CD38^+^ M1 macrophage (gray), and CD163^+^ M2 macrophage (green) infiltration and expression on Day 56 with zoomed-in Z-stack (A and B) and tile-scans (C). (**D**) Immunostaining images of Reca-1^+^ endothelial cells and CD163^+^ M2 macrophages in NHC on Day 14.

**
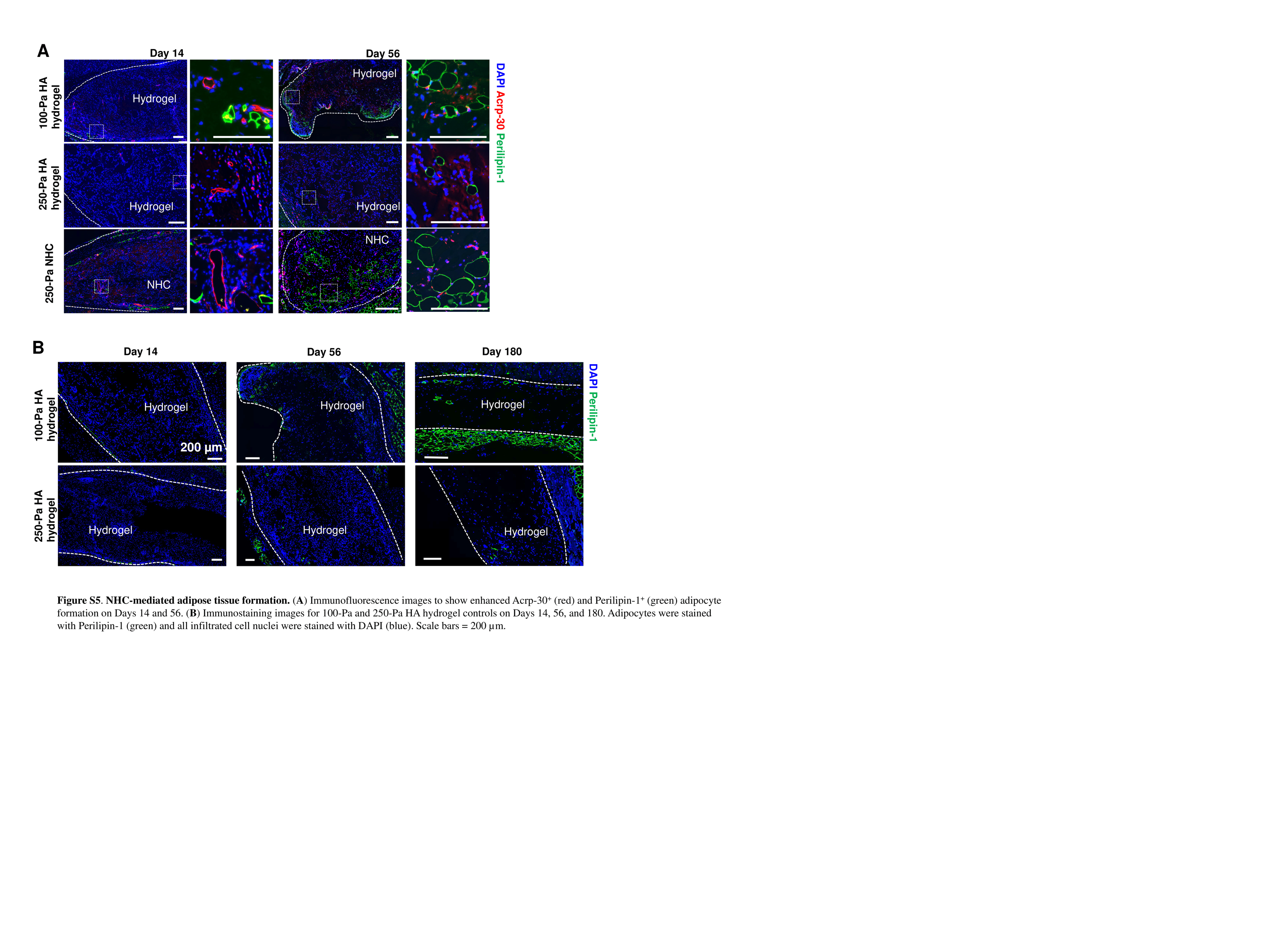
**

**Supplementary Figure S7**. **NHC-mediated adipose tissue formation.** (**A**) Immunofluorescence images to show enhanced Acrp-30^+^ (red) and Perilipin-1^+^ (green) adipocyte formation on Days 14 and 56. (**B**) Immunostaining images for 100-Pa and 250-Pa HA hydrogel controls on Days 14, 56, and 180. Adipocytes were stained with Perilipin-1 (green), and all infiltrated cell nuclei were stained with DAPI (blue). Scale bars = 200 µm.

**
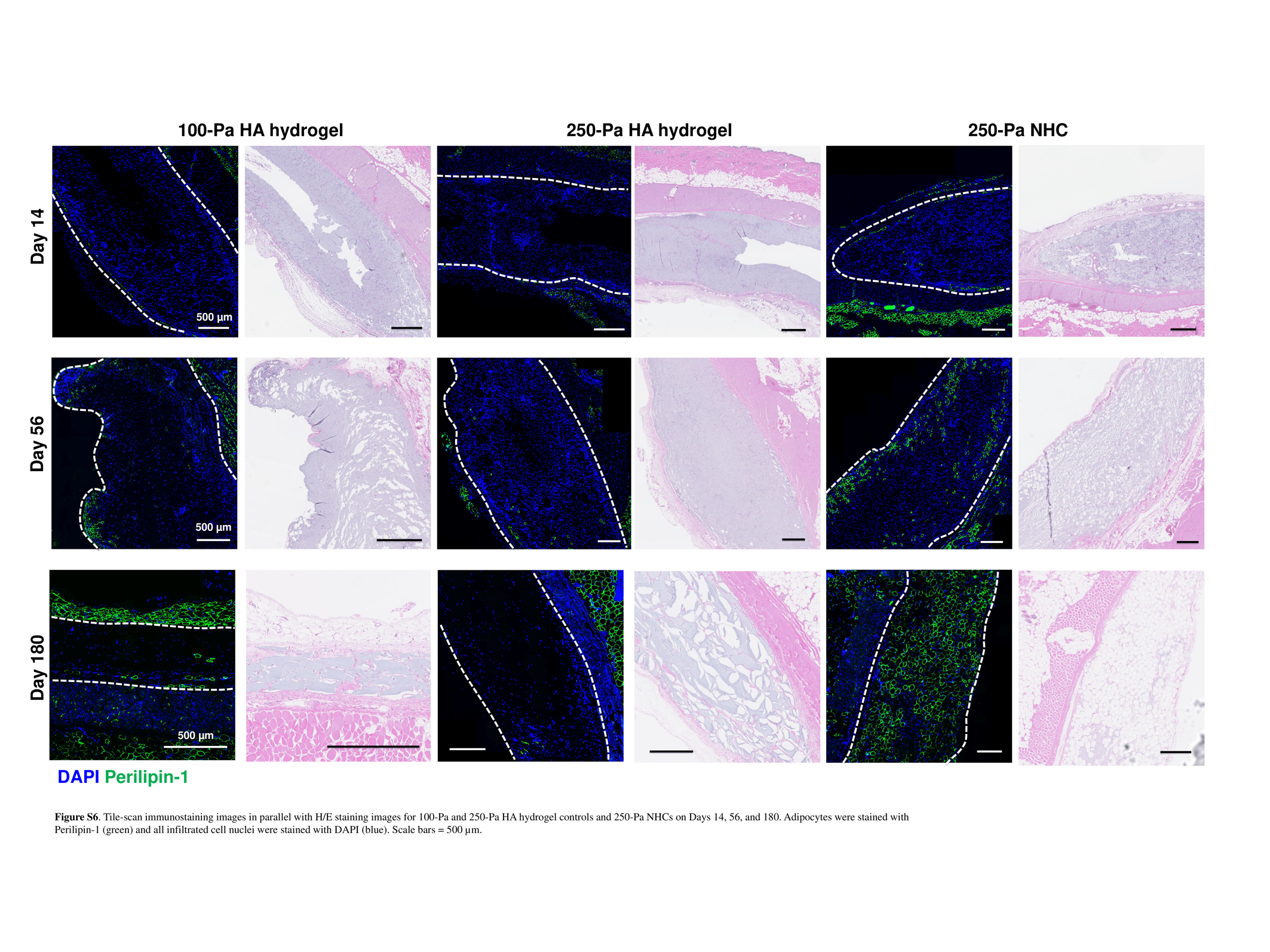
**

**Supplementary Figure S8**. Tile-scan immunostaining images in parallel with H/E staining images for 100-Pa and 250-Pa HA hydrogel controls and 250-Pa NHCs on Days 14, 56, and 180. Adipocytes were stained with Perilipin-1 (green), and the nuclei of all infiltrated cells were stained with DAPI (blue). Scale bars = 500 µm.


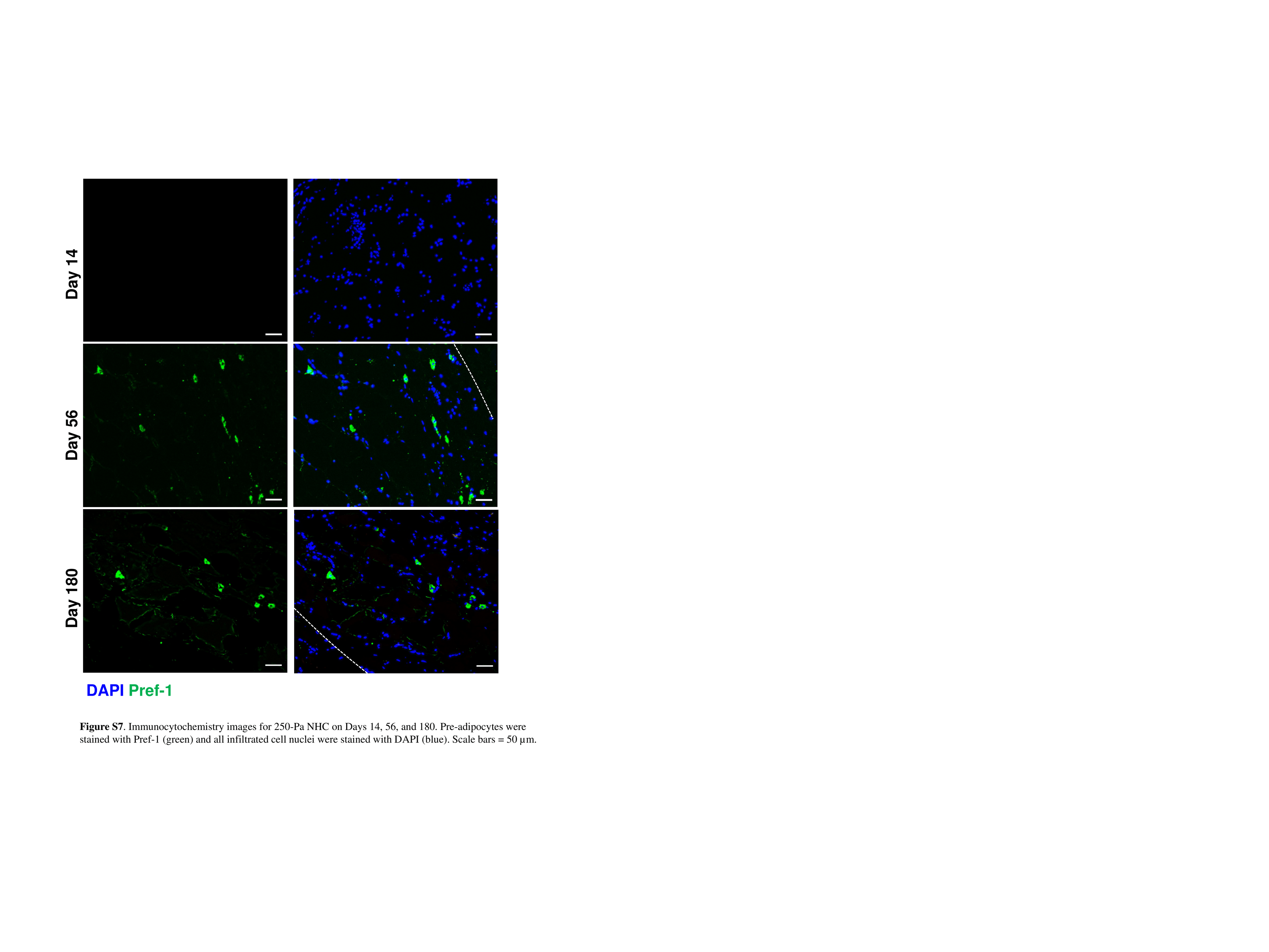


**Supplementary Figure S9**. Immunocytochemistry images for 250-Pa NHC on Days 14, 56, and 180. Pre-adipocytes were stained with Pref-1 (green), and all infiltrated cell nuclei were stained with DAPI (blue). Scale bars = 50 µm.

**Table S1**. Primary antibodies used in this study.

| **Antibody Target** | **Company** | **Catalog Number** |
| --- | --- | --- |
| CD68 | Abcam | ab31630 |
| CD38 | GeneTex | GTX37752 |
| CD163 | Abcam | ab182422 |
| αSMA | Abcam | ab5694 |
| Reca-1 | Abcam | ab9774 |
| Acrp-30 | Bio-Techne | AF3100 |
| Perilipin-1 | Abcam | Ab3526 |
| PDGFR-α | Novus | NBP2-67025 |
| Pref-1 | Biorbyt | orb640782 |

**Table S2**. Secondary antibodies used in this study.

| **Antibody** | **Company** | **Catalog Number** |
| --- | --- | --- |
| Cy3 conjugation,  Donkey anti-mouse | Jackson ImmunoResearch Laboratories Inc. | 715-165-151 |
| Cy3 conjugation,  Donkey anti-goat |  | 705-165-147 |
| Cy5 conjugation,  Donkey anti-rabbit |  | 711-175-152 |
